# Cavin-3 Knockout Mice Show that Cavin-3 Is Not Essential for Caveolae Formation, for Maintenance of Body Composition, or for Glucose Tolerance

**DOI:** 10.1371/journal.pone.0102935

**Published:** 2014-07-18

**Authors:** Libin Liu, Carsten G. Hansen, Brian J. Honeyman, Benjamin J. Nichols, Paul F. Pilch

**Affiliations:** 1 Department of Biochemistry, Boston University School of Medicine, Boston, Massachusetts, United States of America; 2 Laboratory of Molecular Biology, Medical Research Council, Cambridge, Cambridgeshire, United Kingdom; 3 Department of Medicine, Boston University School of Medicine, Boston, Massachusetts, United States of America; University of British Columbia, Canada

## Abstract

The cavins are a family of proteins associated with caveolae, cavin-1, -2 and -3 being widely expressed while cavin-4 is restricted to striated muscle. Deletion of *cavin-1* results in phenotypes including metabolic changes consistent with adipocyte dysfunction, and caveolae are completely absent. Deletion of *cavin-2* causes tissue-specific loss of caveolae. The consequences of *cavin-3* deletion are less clear, as there are divergent data on the abundance of caveolae in *cavin-3* null mice. Here we examine the consequences of cavin-3 deficiency *in vivo* by making *cavin-3* knockout mice. We find that loss of cavin-3 has minimal or no effects on the levels of other caveolar proteins, does not appear to play a major role in formation of protein complexes important for caveolar morphogenesis, and has no significant effect on caveolae abundance. *Cavin-3* null mice have the same body weight and fat mass as wild type animals at ages 8 through 30 weeks on both normal chow and high fat diets. Likewise, the two mouse strains exhibit identical glucose tolerance tests on both diets. Microarray analysis from adipose tissue shows that the changes in mRNA expression between *cavin-3* null and wild type mouse are minimal. We conclude that cavin-3 is not absolutely required for making caveolae, and suggest that the mechanistic link between cavin-3 and metabolic regulation remains uncertain.

## Introduction

Caveolae were first defined by their appearance as “little caves” that were observed in electron micrographs of cell surfaces [1], [2]. These cup-shaped invaginations of the plasma membrane are expressed in many cell types and are most abundant in adipocytes, endothelial and smooth muscle cells (reviewed in [3], [4], [5]). Caveolae have been suggested to play a role in a variety of pathologies, including cancer, diabetes, and cardiovascular disease and loss of caveolae causes muscular and lipodystrophies, insulin resistance and cardiovascular defects amongst a variety of other abnormalities (reviewed in [5]). Recently it has been suggested caveolae also plays a role as mechanosensor [6]. Caveolin-1 (Cav1) was the first protein discovered that is necessary for forming the caveola structure [7]. Cavins-1-4 are another group of proteins that have been documented recently as being caveolar components [8], [9], [10], [11], [12]. Cavin-1 was first described as polymerase I transcription release factor (PTRF) [13] and this protein may function in this role independently of caveolae [14]. Cavin-2 was discovered as a serum deprivation response gene (SDPR [15] and cavin-3 was named SRBC for SDPR-related gene that binds PKC [16] or protein kinase C, delta binding protein (prkcdbp). Cavin-4 is also known as muscle-related coiled-coil protein (MURC) and is found only in cardiac and skeletal muscle [17].

A *cavin-1* null mouse confirmed *in vitro* [10] and zebra fish data [8] documenting that its expression is an absolute requirement for caveolae structure, and the mouse study also revealed a distinct lipodystrophic, insulin resistant phenotype [9], one subsequently documented in humans deficient in this protein [5], [18]. Cavin-2 deficiency in mice causes a tissue-specific loss of caveolae affecting lung and adipose endothelium, but not the endothelium of skeletal and cardiac muscle [19]. The physiological consequences of the cavin-2 loss *in vivo* are currently under investigation. *In vitro*, cavin-2 knockdown causes loss of caveolae [11], [12], [20], induces membrane curvature in caveolae [12], and serves as part of a protease-sensitive cholesterol sensor in caveolae whereby cavin-2 resynthesis restores cholesterol and relocalizes cavin-1 from the cytosol to the plasma membrane [20].

Cavin-3 deficiency in mice as reported by Hansen *et al* was without apparent effect on caveolae abundance in the endothelium of lung, heart, skeletal muscle and adipose tissue, and in the adipocytes of abdominal fat [19]. As is the case for cavin-1 & -2, immuno-electron microscopy and co-immunoprecipitation have shown that cavin-3 localizes to caveolae and associates with the other caveolae proteins [11], [21]. An *in vitro* study showed that the absence of cavin-3 caused defects in caveolae trafficking, possibly by the lack of association with microtubules in the knockdown [21]. A *cavin-3* knockout study was recently published, which reported that the null mice at 2 years of age had a 40% reduction on their body weight and severe lipodystrophy [22], and that knockdown of cavin-3 expression using siRNAs in fibroblasts caused loss of caveolae. The uncertainty over the role of cavin-3 in generating caveolae, and in the role of cavin-3 in regulating metabolism, prompted us to examine the phenotype the cavin-3 deficient mice generated independently In Cambridge and in Boston. We observe minimal changes in caveolae number and in the level of cavin-1, and no apparent metabolic changes in mice on normal and high fat diets at 30 weeks of age.

## Results

### Cavin-3 does not play a major role on caveolae formation

**Figure 1:**
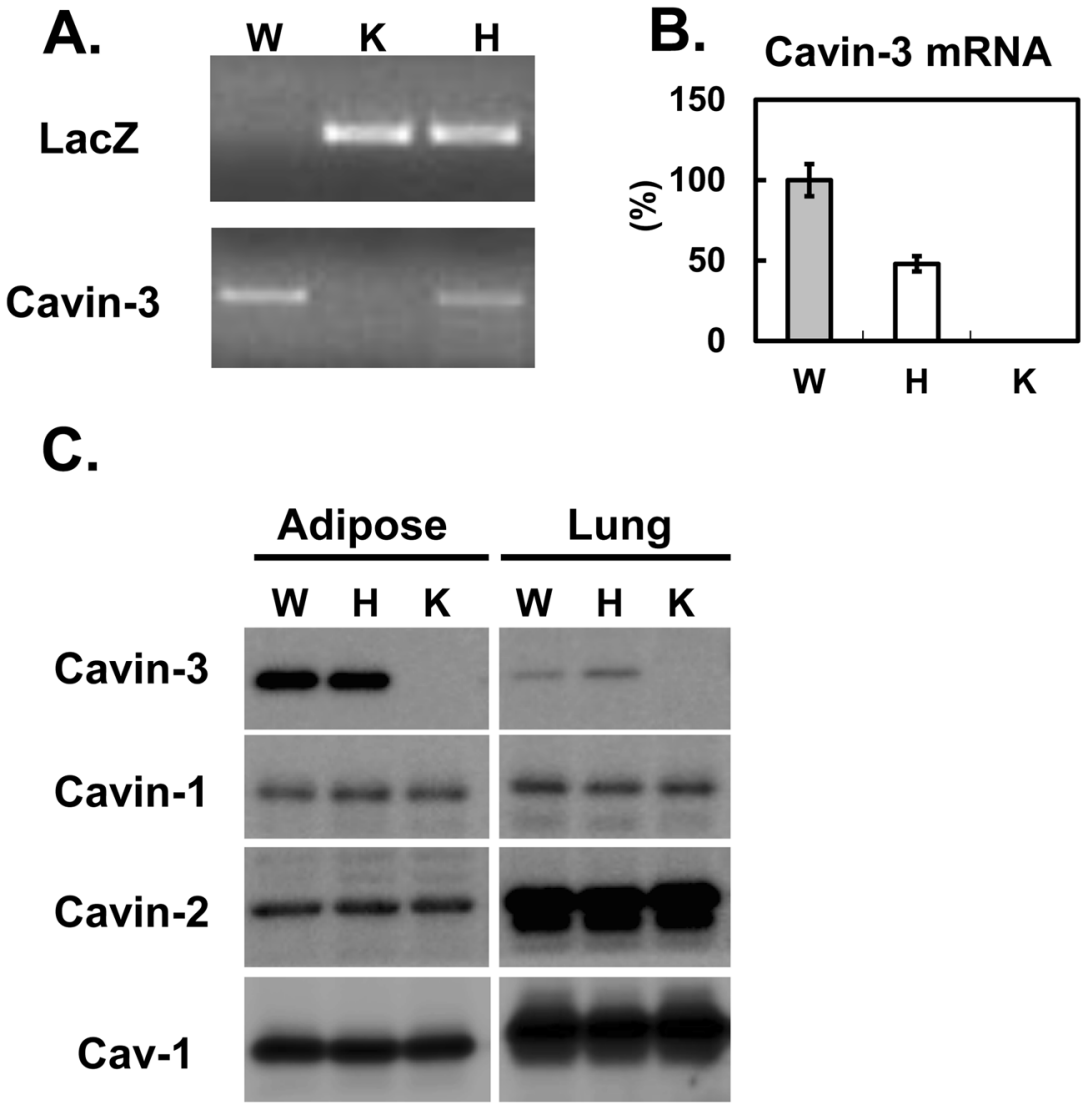
Generation of Cavin-3 knockout mouse. *Cavin-3* knockout mice were generated as described in “Method”. A. PCR genotyping was performed by using primers against LacZ and Cavin-3. B. Adipose tissue lysates (10 µg) from wild type (W), heterozygous (H) and knockout (K) mouse were separated by SDS-PAGE and transferred to PVDF membrane for immunoblotting analysis using the antibodies indicated.

**Figure 2:**
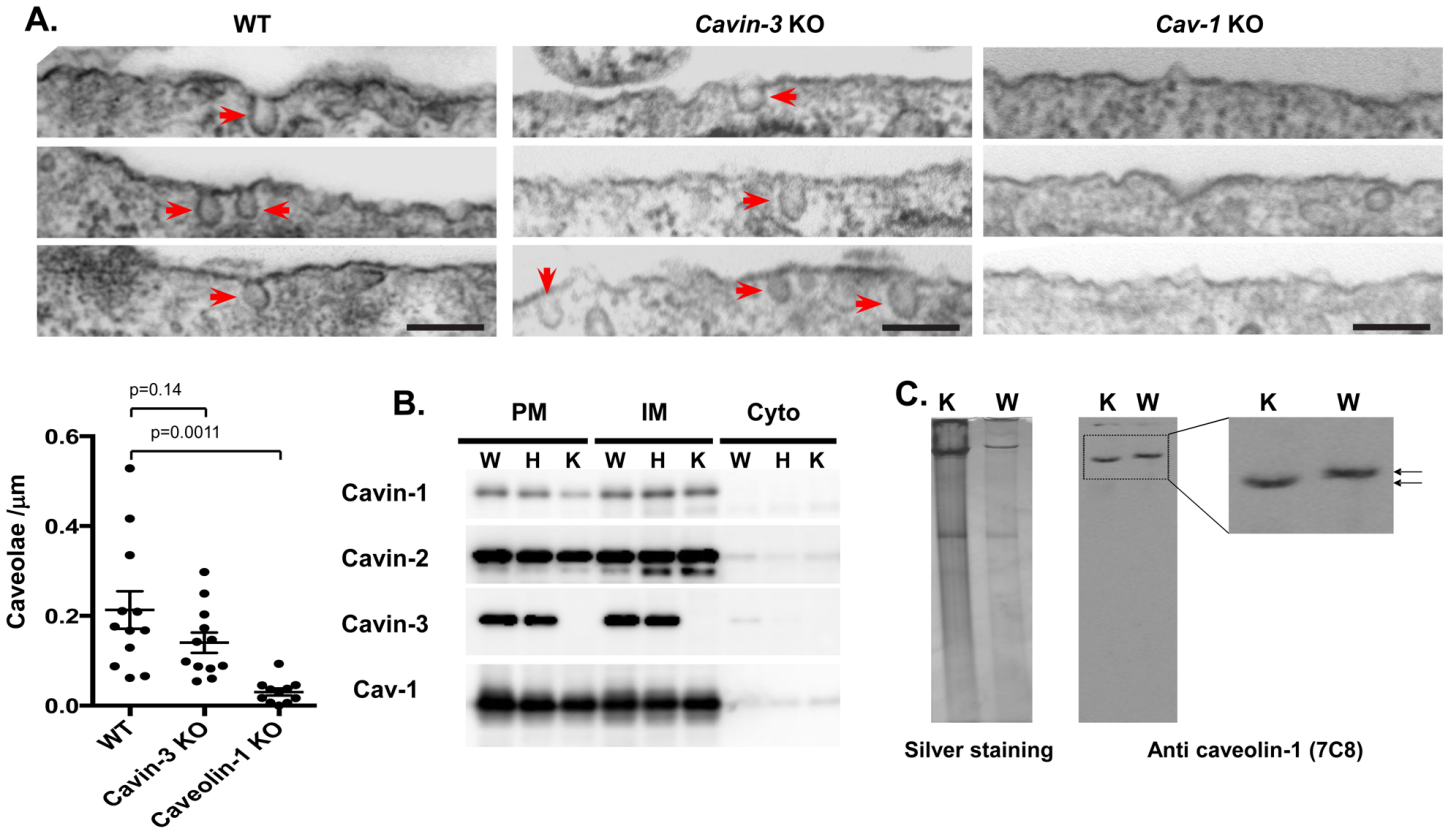
Loss of cavin-3 does not affect the expression of other caveolar proteins, their localization, or the assembly of high molecular weight caveolin complexes. A. Abundance of caveolae in Cavin-3, Cav1 knockout and WT MEFs cells. Multiple electron micrographs were obtained for each cell type (396 of control cells and 250 of cavin-3 KO cells), and both the number of caveolae and total length of plasma membrane present were quantified in each image. Caveolae were counted as omega shaped membrane profiles open at the cell surface. Bars are SEM, p values were calculated using an unpaired t-test. B. Subcellular fractionations (PM: Plasma membrane, IM: internal membranes, Cyto, cytosol) of adipocytes from wild type (W), heterozygous (H) and knockout (K) was performed as described in *Materials and Methods*. Equal protein amounts of the fractions were separated by SDS-PAGE and analyzed by Western blot by using the antibodies to the proteins indicated. Detection was by enhanced chemiluminescence (ECL). C. Wild type (WT) and cavin-3 knockout (KO) adipose tissues were solubilized in 1 ml of MBS containing 1% (v/v) Triton X-100. The lysate was homogenized and mixed with an equal volume of 80% sucrose in MBS (final volume, 2 ml) and overlaid successively with 2 ml of 30% sucrose and 1 ml of 5% sucrose (in MBS). After centrifugation at 200,000 *g* for 18 h, 0.4-ml fractions were collected from the bottom of the gradient (*fraction 1* is the top fraction). Lipid raft fractions were separated in native PAGE followed by silver staining or transferred to PVDF membrane for immunoblotting analysis using the antibodies indicated.

*Cavin-3* knockout mice were generated independently (see Methods) using targeted ES cells (KOMP, VD114488) where the full length *Cavin-3* gene is replaced by a lacZ/neo fusion cassette. Analyses of genomic DNA by PCR ([19] & Figure 1A), RNA by RT-qPCR, (Figure 1B), and protein by western blot (Figure 1C), confirmed the gene disruption and absence of cavin-3 protein in mice homozygous for the targeted allele (K) (also see Fig. 1 in [19]). The expression of the other abundant caveolar proteins, cavin-1 & -2 and caveolin-1 (Cav1) is unchanged. These data independently confirm those of Hansen *et al.* on caveolar protein expression in cavin-3 null mice [19]. Next we checked the abundance of caveolae structures using electron microscopy (EM) in mouse embryonic fibroblasts (MEFs) derived from the knockout. As shown in Figure 2A, there is no statistically significant difference between wild type and *Cavin-3* knockout, although there is a trend towards lower caveolae number in the knockout. In addition, caveolae in the *cavin-3* knockouts are not visibly different in morphology from those in control cells (Figure 2A). In contrast and as expected, the EM of Cav1 null MEFs shows a virtual absence of morphological caveolae, implying that the morphological structures identified as caveolae in *cavin-3* null MEFs indeed represent these specific domains. By using a standard fractionation protocol, we show there is no change in the amount of other caveolae proteins such as cavin-1, -2 and Cav1 (Figure 1B) and in their subcellular distribution in adipocytes (Figure 2B). The adipocyte has a major contribution to the lipodystrophic phenotype of *Cav1* [23] and *cavin-1* [9], [19] knockout mice. From the data of Figures 1 and 2, we conclude cavin-3 does not play a major role on caveolae formation, a result consistent with the previous report [19].

### Loss of cavin-3 caveolae minimally alters cavin complex integrity

To further examine if cavin-3 plays any role in caveolae complexes, we purified lipid raft fractions from adipose tissue using an established protocol based on the detergent resistance and the low buoyant density of lipid raft to separate caveolae fractions from other membrane proteins. Subsequently the protein complex(es) from lipid raft fractions was separated by a 5% native gel followed by silver staining or transferred to a PVDF membrane followed by western blotting using anti-cav1 (7C8) antibody. As shown in Figure 2C, a caveolin complex was identified at a high molecular weight (ca.≥500 KD), and interestingly, the complex from cavin-3 knockout mice shows a slight but significantly faster migrating band comparing to wild type complexes. This slight increase in mobility is consistent with loss of one component of the complex (cavin-3), but unaltered integrity of the complex *per se*. This result, together with previously published analysis of cavin complexes in *cavin-3* knockouts on sucrose velocity gradients [19], suggests cavin-3 is unlikely to be essential for the assembly of other caveolar components into high molecular weight complexes.

### Loss of cavin-3 does not have significant impact on adipose tissue and glucose metabolism

**Figure 3:**
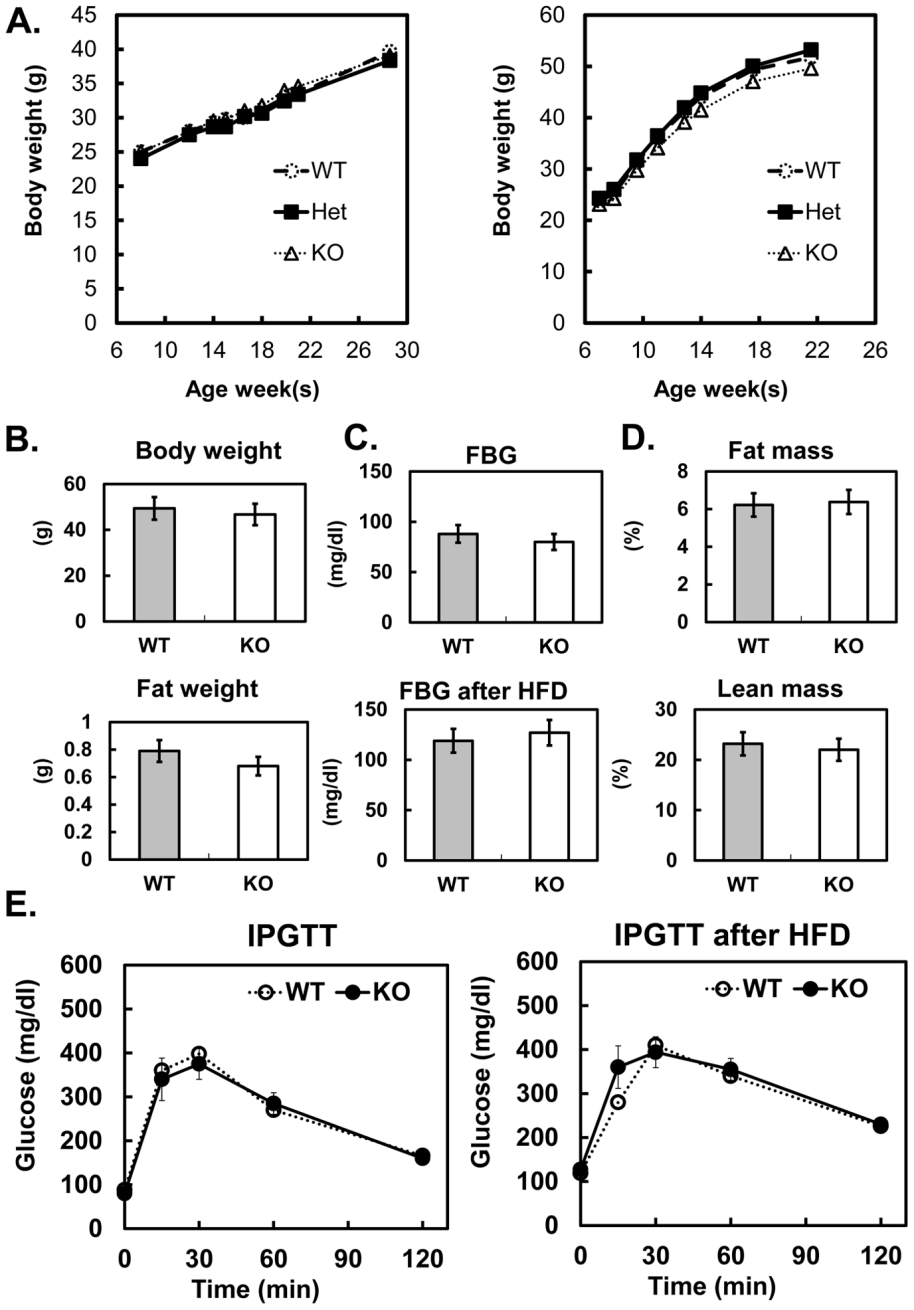
No metabolic phenotypes were detected in Cavin-3 null mice. Cavin-3 null and wild type male mice (n = 8 per group) were given a high fat diet (HFD) starting at 8 weeks of age. The mice fed normal chow served as a control. After 14 weeks on HFD, the animals were fasted for 4–6 hours and the metabolic phenotypes were measured. The body weight gain curve from control and HFD (A), fasting body and fat tissue weight(B), fasting blood glucose levels of control and after HFD (C), body composition (D) and Intraperitoneal glucose tolerance test (IPGTT) (E) are shown.

Mice lacking Cav1 and cavin-1 protein have markedly reduced adipose tissue mass and major metabolic dysfunctions due, in part, to the absence of caveolae in adipose tissue [9], [19], [23], [24]. These functional deficiencies include dyslipidemia and impaired glucose tolerance as well as resistance to weight gain on a high fat diet. Thus we determine growth curves for WT, *cavin-3* heterozygous and *cavin-3* null mice and we found them to be identical over a 30 weeks period (Fig. 3A). Upon high fat diet feeding, *cavin-3* null mice gain weight to the same degree as wild type mice. There is no difference in the fat to lean mass ratio and in fat tissue weight between WT and KO. To assess insulin sensitivity, we measured fasting blood glucose and performed intra-peritoneal glucose tolerance tests (IPGTTs) on both normal and high fat fed mice of both genotypes, and we see no differences between *Cavin-3* null and WT mice.

**Figure 4:**
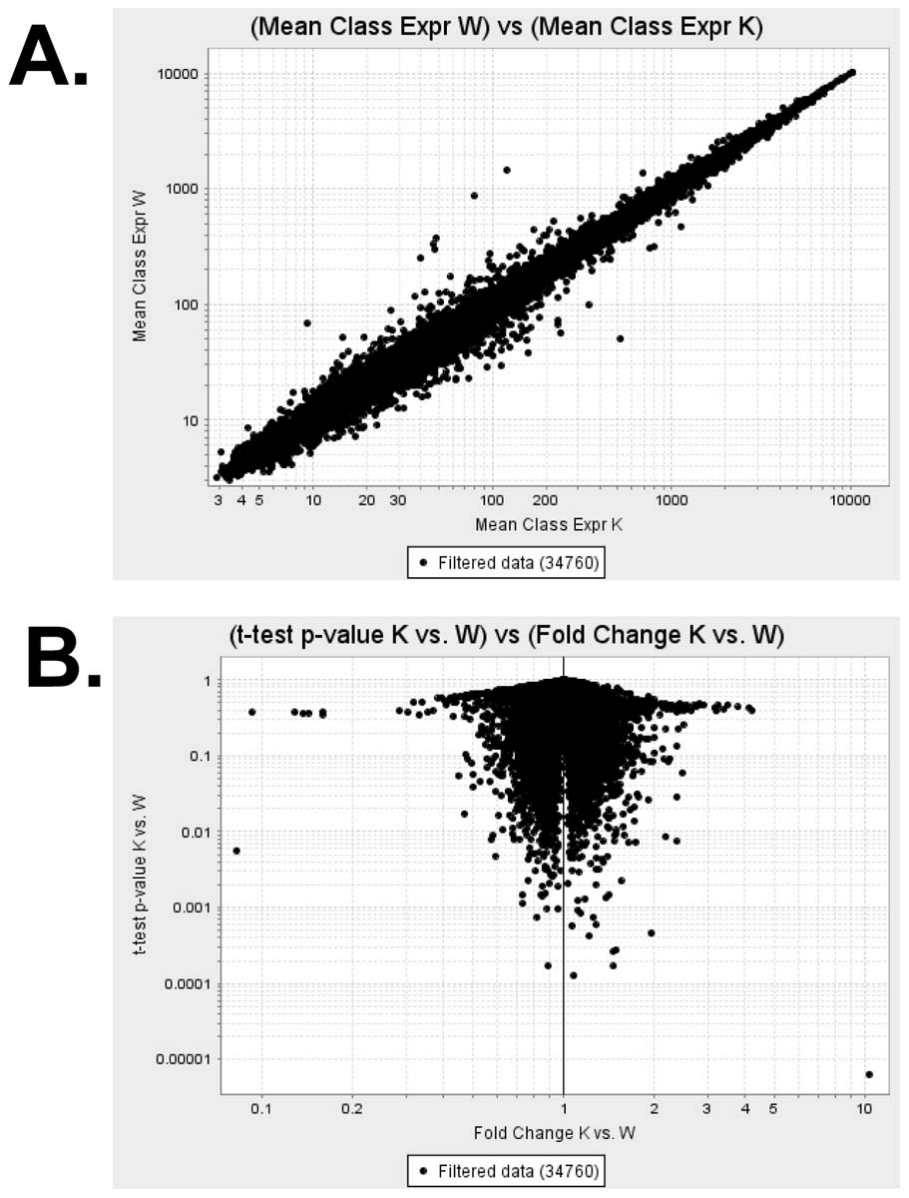
Microarray analysis from adipose tissue shows minimum changes between cavin-3 null and wild type mice. Total RNA was isolated using Trizol (Invitrogen). After checking the sample integrity total RNA was reverse transcribed and labeled with biotin. The labeled, fragmented DNA was hybridized to the Mouse Gene 1.0 ST Array. Thereafter, the data were processed, normalized, analyzed, and visualized by GenePattern software modules. The results are shown in A. mean value from wild type and cavin-3 null samples (n = 3 mice, each genotype) and B. Volcano plots.

To further explore the possible changes due to the loss of cavin-3 in adipose tissue, we performed microarray analysis in adipose tissue from WT and KO mice (n = 3, each genotype). As shown in the Volcano plot of Figure 4, the changes between KO and WT are minor, and the genes which change by >2-fold or <0.5 fold with P value smaller than 0.01 are limited. As shown in Table 1, the altered mRNAs have no obvious connection to adipocyte or caveolar biology. As expected, cavin-3 mRNA is the most dramatically down-regulated in the null mice and none of the other caveolar genes is affected by *Cavin-3* deletion (Table 1). In addition, a pathway analysis did not show any significant changes, null *versus* WT (data not shown).

**Table 1:** List of top down and up-regulated and caveolae related genes in microarray.

Entrez Gene ID	Symbol	Description	KO/WT (Folds)
Down			
109042	Prkcdbp	protein kinase C, delta binding protein	0.08
109637	Upk1a	uroplakin 1A	0.33
18436	P2rx1	purinergic receptor P2X, ion channel, 1	0.51
13074	Cyp17a1	cytochrome P450, family 17, subfamily a1	0.52
17068	Ly6d	lymphocyte antigen 6 complex, locus D	0.54
22269	Upk2	uroplakin 2	0.57
104112	Acly	ATP citrate lyase	0.63
11468	Actg2	actin, gamma 2, smooth muscle, enteric	0.64
100101484	Trac	T cell receptor alpha constant	0.66
12797	Cnn1	calponin 1	0.66
20250	Scd2	stearoyl-Coenzyme A desaturase 2	0.66
110310	Krt7	keratin 7	0.67
22142	Tuba1a	tubulin, alpha 1A	0.67
83433	Trem2	triggering receptor expressed on myeloid cells 2	0.67
Up			
244189	Gm4972	predicted gene 4972	3.34
21743	Inmt	indolethylamine N-methyltransferase	2.76
386463	Cdsn	corneodesmosin	2.56
11657	Alb	albumin	2.33
29818	Hspb7	heat shock protein family, member 7	1.86
11571	Crisp1	cysteine-rich secretory protein 1	1.86
18546	Pcp4	Purkinje cell protein 4	1.83
54612	Sfrp5	secreted frizzled-related sequence protein 5	1.71
213742	Xist	inactive X specific transcripts	1.68
17393	Mmp7	matrix metallopeptidase 7	1.67
20701	Serpina1b	serine preptidase inhibitor, clade A, member 1B	1.66
Caveolae related			
19285	Ptrf (Cavin-1)	polymerase I and transcript release factor	0.99
20324	Sdpr (Cavin-2)	serum deprivation response	1.02
12389	Cav1	caveolin 1, caveolae protein	0.99
12390	Cav2	caveolin 2	0.99
259300	Ehd2	EH-domain containing 2	0.98
11754	Aoc3 (SSAO)	amine oxidase, copper containing 3	0.99
20778	Scarb1 (CD36)	scavenger receptor class B, member 1	1.01

## Discussion

Our results clearly show that upon the loss of cavin-3, there is no major impact on the expression of other caveolae proteins such as Cav1, cavin-1 and 2 at both the mRNA (Table 1) and protein levels (Figure 1B, 2B and [19]). Likewise there is no major impact of cavin-3 deficiency on caveolae formation (Fig. 2A, [19]), or the assembly of caveolar proteins into high molecular weight complexes (Fig. 2B, [19]). Body weight and composition (Fig. 3A–D) and glucose tolerance (Fig. 3E) are essentially the same in congenic control and *cavin-3* knockout mice Previous studies also showed that deletion of *cavin-3* resulted in unchanged caveolae number in fat endothelium and in the adipocytes themselves as well as in other tissues [19].

Based on the immuno-E.M staining of cavin-3 in adipocyte caveolae [11] it appears that cavin-3 protein is considerably less abundant than the other cavins, particularly in adipocytes, but it is also less abundant in Hela cells [25]. Of course, it cannot be ruled out that the former result may be due to epitope masking, but taken together, the available stoichiometric and structural data suggest the relative contribution of cavin-3 to the protein complexes of caveolae is the least of the three cavins. Cavin-3 is well conserved across vertebrate species as are other cavin proteins, and so it is likely to be under continued selective pressure. The function of cavin-3 is, however, still unclear. Intriguingly, there is evidence that cavin-3 is involved in circadian rhythm formation, possibly acting outside of caveolae [26].

A recent report from Hernandez *et al.* showed a dramatic phenotype due to *cavin-3* deficiency, with extensive changes in signal transduction proteins in lung at a relatively young age and severe lipodystrophy leading to a 40% body weight reduction at 2 years of age [22]. The metabolic differences can possibly be explained by age, and although we did not examine lung signal transduction, the normal metabolic responses suggest Akt-dependent signaling in insulin responsive tissues is normal. The data generated herein were duplicated using 2 different clones from KOMP to generate ES cells in the C57BL/6 mouse genetic background. In contrast, the genetic background of the ES cells used by Hernandez *et al.* was the 129Sv/J mouse, which was subsequently backcrossed for 8 generations in the C57BL/6 lineage. Their knockout strategy was also different from ours and their genotyping data was not provided [22]. In addition, there is no clear, known function of genes or elements around cavin-3 gene locus. But genetic mouse strain differences can cause significant phenotypic differences [27]. However, we can state with confidence that the mice generated as part of our study lack cavin-3 protein, and this does not result in profound effects on caveolae structure and composition, or on metabolic phenotypes up to age 30 weeks, so cavin-3 is unlikely to be absolutely essential for caveolae and metabolic regulation. It is certainly possible nevertheless, that the *cavin-3* null mice may have a subtle phenotype that will require additional scrutiny to identify.

## Materials and Methods

### Ethics Statement

All animal studies were performed in accordance with the guidelines and under approval of the Institutional Review Committee for the Animal Care and Use of Boston University. Animal use in Cambridge was carried out under a license from the UK Home Office and was approved by MRC-LMB Ethical Review.

### Reagents

Monoclonal antibodies recognizing cavin-1 (2F11) [28] and Cav1 (7C8) [29] have been previously described. The following antibodies were commercially acquired: anti-Cav1 was from BD Transduction Laboratories (San Jose, CA), anti-actin was from Sigma; anti-transferrin receptor was from Zymed Laboratories Inc. Invitrogen. Additional anti-cavin-1/PTRF antibodies were purchased from BD Transduction. Polyclonal rabbit anti-cavin-2/3 antibodies were produced against a peptide sequence at the C terminus of the proteins (21st Century Biochemicals, Hopkinton, MA).

### Mice

The Cavin-3 knockout mice were generated by microinjecting targeted ES cells (KOMP, derived from C57BL/6N, VD11448, clone 11448A-D8 in Cambridge and clone 11448A-A12 in Boston) into C57BL/6 blastocysts, and these gave rise to male chimeras with significant ES cell contribution (as determined by coat color). By mating with C57BL/6 females and genotyping the offspring by PCR analysis, germ line transmission was confirmed. Male and female heterozygous F1 animals were interbred to obtain Cavin-3 knockout (Cavin-3 KO) animals. The MEF experiments of Figure 2 used mice from Cambridge, and all other experiments shown were done using mice from Boston. Male animals only were analyzed at ≥8 weeks of age. The animals were maintained in a pathogen-free animal facility at 21°C under a 12-h light/12-h dark cycle with access to a standard rodent chow. In the feeding studies the mice were fed normal chow (NC, 10% fat, D12450B, Research Diets, Inc.) or a high fat diet (HFD, 60% fat, D12492, Research Diets, Inc.). Except when specifically noted, all mice used for *in vivo* or *in vitro* studies were fasted for 4–6 hours starting from early morning prior to use. For tissue harvesting, mice were sacrificed under CO2 anesthesia, and tissues were rapidly taken and immediately frozen in liquid nitrogen and stored at −80°C until biochemical analysed.

The cavin-3 knockout mice produced in Cambridge have been previously published [19], and were derived from KOMP clone 11448A-D8. Mouse embryo fibroblast (MEF) were produced from these mice by timed mating and embryo harvest at day E12.

### Subcellular fractionation and lipid raft flotation

This procedure was performed on mouse adipocytes essentially as originally described for rat adipocytes [30]. Isolated mouse adipocytes were washed by cold PBS for three times, cold HES buffer once, and homogenized with a Teflon-glass tissue grinder in HES buffer. Subcellular fractions (plasma membrane (PM), internal membranes (IM) and cytosol (Cyto)) were obtained by differential centrifugation and resuspended in HES. Buffers used with subcellular fractionation contained a protease inhibitors cocktail from Sigma. The flotation protocol was performed as described in previous studies [10]. Briefly, 0.5 g adipose tissue were lysed in 2 ml of MBS (25 mM MES and 150 mM NaCl, pH 6.5) containing 1% Triton X-100 and supplemented with a protease inhibitor mix (Roche Molecular Biochemicals). The samples were then incubated at 4°C for 20 min with end-over-end rotation. The solubilized lysates were homogenized with 10 strokes of a Dounce homogenizer, and 1 ml of the homogenate was added to an equal volume of 80% (w/v) sucrose in MBS. The solubilized cells (in 40% sucrose) were placed at the bottom of a centrifuge tube and overlaid successively with 2 ml of 30% sucrose and 1 ml of 5% sucrose (in MBS). After centrifugation at 240,000×g in a Beckman SW55 rotor for 18 hours, 0.3- to 0.4-ml fractions were collected from the bottom of the gradient.

### Gel Electrophoresis and immunoblotting

Proteins were resolved by SDS-PAGE as described [31]. Gels were transferred to PVDF (polyvinylidene difluoride) membranes pretreated with methanol (Biorad, Hercules, CA) in 25 mM Tris, 192 mM glycine. Membranes were blocked with 1% BSA in PBS containing 0.1% Tween 20 for 1 hour at room temperature. Membranes were then probed with the primary antibodies for either overnight at 4°C or 2 hours at room temperature and incubated with Horseradish peroxidase-conjugated secondary antibodies (Sigma). Signals were enhanced with chemiluminescence reagents (Perkin Elmer Life Sciences, Boston, MA) for detection of Western signals using a Fujifilm LAS-4000 scanner or Autoradiography Film (Molecular Technologies, St. Louis MS).

### Microarray analysis

All procedures were performed at the Boston University Microarray Resource Facility. Briefly, the total RNAs from 3 each WT and cavin-3 KO were isolated using Trizol (Invitrogen) and sample integrity was verified using RNA 6000 Pico Assay RNA chips run in Agilent 2100 Bioanalyzer (Agilent Technologies, Palo Alto, CA). Total RNA was reverse transcribed using Ovation Pico WTA System V2 (Nugen, San Carlos, California). The obtained SPIA-amplified cDNA was purified using Agencourt RNA clean XP Purification Beads and fragmented (5 ng) and labeled with biotin using the Encore Biotin Module (NuGEN, San Carlos, California). SPIA-amplified cDNA and fragmented cDNA quality controls were carried out by running an mRNA Pico assay in the Agilent 2100 Bioanalyzer. The labeled, fragmented DNA was hybridized to the Mouse Gene 1.0 ST Array (Affymetrix, Santa Clara, CA) for 18 hours in a GeneChip Hybridization oven 640 at 45°C with rotation (60 rpm). The hybridized samples were washed and stained using an Affymetrix fluidics station 450. After staining, microarrays were immediately scanned using an Affymetrix GeneArray Scanner 3000 7 G Plus (Affymetrix, Santa Clara, CA). Thereafter, the data were processed, normalized, analyzed, and visualized by GenePattern software modules. The dataset has been submitted to Gene Expression Omnibus (GEO) and the accession number is GEO57513.

### Electron microscopy and quantification of caveolar abundance

MEFs were fixed with 2.0% PFA, 2.5% Glutaraldehyde (GA) in 0.1 M Sodium Cacodylate buffer pH 7.4. Following several buffer washes, cells were post fixed in 1% Osmium Tetroxide in 0.1 M Sodium Cacodylate buffer for 1 hour at 4°C. They were then *enbloc* stained with 2% Uranyl Acetate in 30% ethanol and further dehydrated in an increasing ethanol series, followed by propylene oxide and infiltration and embedding in CY212 resin. Sections were counterstained with Reynolds Lead Citrate and viewed on a Philips 208 microscope operated at 80 kV. Digital images were obtained using a CCD camera. Caveolae were quantified by tracing the perimeter of entire cell outlines from images at 36000×magnification (15–40 images per cell), and counting the caveolae in each image by morphology. The investigator doing the counting was blind to the genotype of the sample. 12 randomly selected cells were analysed per genotype.

### Statistical analyses

Data are presented as the means ± SEM. The significance of differences between groups was evaluated using a student t-test. A p value <0.05 was considered significant.
